# Exploring the Antimicrobial Potential and Stability of Black Soldier Fly *(Hermentia illucens)* Larvae Fat for Enhanced Food Shelf-Life

**DOI:** 10.3390/gels9100793

**Published:** 2023-10-02

**Authors:** Aelita Zabulionė, Alvija Šalaševičienė, Natalja Makštutienė, Antanas Šarkinas

**Affiliations:** Food Institute, Kaunas University of Technology, LT-50254 Kaunas, Lithuania; alvija.salaseviciene@ktu.lt (A.Š.); natalja.makstutiene@ktu.lt (N.M.); antanas.sarkinas@ktu.lt (A.Š.)

**Keywords:** Black Soldier Fly (*Hermetia illucens*) larvae, larval fat, antimicrobial activity, model food matrix, oleogel from larval fat

## Abstract

The larvae of the Black Soldier Fly (BSF, *Hermetia illucens*) have been introduced as one of the tools to create a circular economy model, which will be used in areas such as waste management and the treatment of industrial by-products to produce high-added-value food grade ingredients. The main aim of this research was to investigate the fat composition and antimicrobial activity against food pathogens and spoilers of Black Soldier Fly larvae. The research revealed that the Black Soldier Fly larvae fats are predominantly lauric fatty (40.93%), which are followed by palmitic, oleic, myristic, linolenic and palmitoleic fatty acids, accounting for 19.11, 17.34, 6.49, 8.79 and 3.89% of the fatty acid content, respectively. The investigation of the fats showed stability through a one-year monitoring period with no indication of chemical or microbiological spoilage. Different fat fractions were tested for antimicrobial activity, which showed efficiency against *Candida albicans* (the inhibition zone varied from 10.5 to 12.5 mm), *Bacillus subtilis* (from 12.5 to 16.5 mm), *Staphylococcus aureus* (12.5 mm) and *Escherichia coli* (10.0 mm). The inhibitory effect on *Candida albicans* was confirmed by shelf-life studies using larvae fat-based oleogel in a model food matrix. GraphPad Prism (ver. 8.0.1) was used for the statistical data processing. This research revealed the potential of Black Soldier Fly larvae fat as a very stable ingredient with promising antibacterial properties that can extend the product shelf-life in food matrixes even when used in relatively small amounts.

## 1. Introduction

The next few years will be a vital period to exploit the potential of Black Soldier Fly larvae as a waste converter, protein source for feed, and as a source of various biochemicals (e.g., proteins, fats, chitin, etc.). Since 2018, there have been quite a few research studies about the Black Soldier Fly or larvae adaptations for organic waste management by using it as animal feed [1]. This suggests that this theme has great potential, but until now, it has not been investigated as a food ingredient for human consumption. There is a need to focus on the nutritional and technological benefits and the proven chemical and microbiological safety of BSF larval fat in order to avoid an instant psychological rejection on behalf of consumers [2].

In the natural world, many insects primarily feed on animal waste, which they can effectively transform into biomass. One of the most environmentally and commercially sensible insect species is the Black Soldier Fly (BSF, *Hermetia illucens*; Diptera, *Stratiomyidae*). Kitchen waste, damaged feed, manure and other forms of waste can all be converted effectively by BSF larvae [3]. As a result, as it develops from larva to adult, this species comes into touch with dangerous germs, yet it possesses a defence against infection. According to reports, BSF larvae have been found to considerably lower the populations of many zoonotic pathogens in dairy and cattle manure. Additionally, BSF larvae have been found to have several antibacterial compounds and properties. The potent antibacterial properties of their immune system’s haemolymph against three different *Salmonella* species was confirmed in the vaccinated haemolymph of BSF larvae [4].

Like any other living being, the feed provided to Black Soldier Fly larvae has an impact on their composition. However, according to research [5], modifications of polyunsaturated fatty acids in the Black Soldier Fly larvae as a result of their diets may be limited. When their meals were devoid of these fatty acids, the larvae were able to synthesise C12:0, C14:0 and C16:1n-9 in vivo. A quantitative assessment provided some nutritional strategies for better utilising the Black Soldier Fly larvae as a food source, such as using a sufficient quantity of Black Soldier Fly larvae in aquafeed as the primary source of SFA and lauric acid, as well as a partial source of protein, MUFA and PUFA, in order to reduce the need for fish meal and fish oil.

The majority of the antibacterial properties of BSF larvae have been attributed to the soluble peptide fractions that were extracted by acidifying the whole larval body. *Staphylococcus aureus*, methicillin-resistant *S. aureus*, *S. epidermidis*, *Streptococcus pyogenes, Klebsiella pneumoniae, Pseudomonas aeruginosa* and *Escherichia coli* pathogens were the seven selected human wound pathogens in a study where the methanol extract of a whole larval body of *L. cuprina* demonstrated an in vitro antibacterial activity against this extract. The reconstituted larval extract also has excellent tensile strength and thermal stability [6].

Previous studies on the extraction of BSF larval lipids, proteins and chitin have repeatedly focused on a single developmental stage—namely, the larval stage—while in practice, individuals from different life stages can be found in the same rearing batch as larvae, pre-pupae and pupae studies [7].

Food waste should be recycled as animal (including Black Soldier Fly larvae) feed or as compost and not transferred directly to biofuel processing. Various sources have highlighted that the treatment of waste with BSF larvae can have a lower environmental impact than composting [8].

Larval biomass is rich in proteins and lipids, and it is used as a raw material for a variety of applications in food systems. For example, proteins and lipids are being used in feed for livestock, fish, poultry, pigs and pets, while the exoskeleton of the larvae is processed into chitosan. The industrial production of aquaculture feeds (e.g., products of the genus *Schizochytrium*) generates a large amount of waste, which may contain high levels of fatty acids. The BSF larvae fed on *Schizochytrium* production waste have a good bioaccumulation of polyunsaturated fatty acids, which allows them to accumulate omega-3; therefore, they could be used as a sustainable food additive in aquaculture [9].

Insects are considered to be a valuable source of alternative protein from a nutritional point of view, and the efficient isolation of their different fractions is a prerequisite for their large-scale use [10].

The insect *Tenebrio molitor* is also attracting worldwide attention as a source of protein for food. But contrary to *Tenebrio molitor*, the Black Soldier Fly has already been officially announced as suitable for human consumption in many European Union countries [11]. The untreated larvae contain about 15% fat and 20% protein [11,12]. The levels of minerals, vitamins, amino acids and fatty acids they contain have also been analysed [13]. When comparing to other edible insects, freeze-dried yellow mealworm larvae contained approximately 33% of the fat, 51% crude protein and 43% protein in terms of dry matter [14].

Lipid extractions of *Hermetia illucens* larvae are usually performed with hexane. However, the current aim is to replace these harmful solvents. The solvent 2-methyltetrahydrofuran has been found to be able to extract even higher amounts of free fatty acids and valuable phospholipids compared to hexane. Therefore, lipid extractions with 2-MeTHF can be considered as a suitable, more environmentally friendly alternative to hexane extraction [6,14].

Domestic animal feeding studies have shown that the nutritional value of larvae-based feed is equivalent to that of soybean-based feed with no reduction in laying hens. Thus, soybean-based feeds can be completely replaced by fly meal and fly fat [15].

Fat makes up about 15% to 49% of *H. illucens* larvae, making them a rich source of lipids. Plant pathogenic Gram-negative bacteria, such as *Xanthomonas campestris* subsp. *campestris Pantoea agglomerans*, *Dickeya solani Pectobacterium carotovorum* subsp. *carotovorum* and *Pectobacterium atrosepticum*, are inhibited by the fatty acids and their derivatives of AWME of *H. illucens* larval fat. The current work also demonstrates the potential of using *H. illucens* larval fat to create novel and potent natural disinfectants and antibacterial agents in the future [6].

*Salmonella typhimurium* and *Escherichia coli*, as two significant bacterial strains in poultry, have been tested for antibacterial activity using six different dilution levels. The BSF larva extract exhibited a high level of antibacterial activity against *Salmonella typhimurium* and *E. coli* based on the diameter of the inhibitory zone. Additionally, the BSF larval extract contained a significant amount (49.18%) of lauric acid, which is a saturated fatty acid that has been shown to have antibacterial properties. It follows that the BSF larval extract might be considered as a candidate for an antibacterial material [16].

According to reports, a variety of insects create antibacterial compounds on their surface or in their digestive tracts to guard against microbial infection. The larva of the Black Soldier Fly has been used in a variety of fields until recently, including in the bioconversion of livestock manure, the conversion of organic materials, aquatic and monogastric animal feed, and in forensic science for determining the length of a person’s post-mortem period [16].

Insects offer a good nutritional profile for humans, in addition to being used as other feed, but the existence of allergens raises possible risks that need to be further examined. Overall, it has been shown that producing insects has low energy, land use and environmental impact costs [16,17].

Taking into consideration the fact that the Black Soldier Fly larvae has the potential to inhibit or slow down the growth of common food pathogens and spoilers, this quality could be used in food products as a fat substitute, which would give the extra benefit of prolonging the shelf-life by slowing the growth of microorganisms. This property would be most reasonable in relation to high-humidity, short shelf-life products. The choice to use a specific model food matrix was based on the idea that Black Soldier Fly larvae fat can act as a natural preservative in high-moisture, refrigerated products, since this type of product usually has a short shelf-life or includes some kind of preservative. For investigation purposes, a simple plant-based matrix was selected and prepared from a pea texturate, pea fibre, rapeseed oil and water. This formulation gives the impression of a high-moisture product, which at the beginning, it has low levels of microbiological indicators and has the capacity to support growth (it has high amount proteins as well as a decent amount of carbohydrates and water).

**Figure 1:**
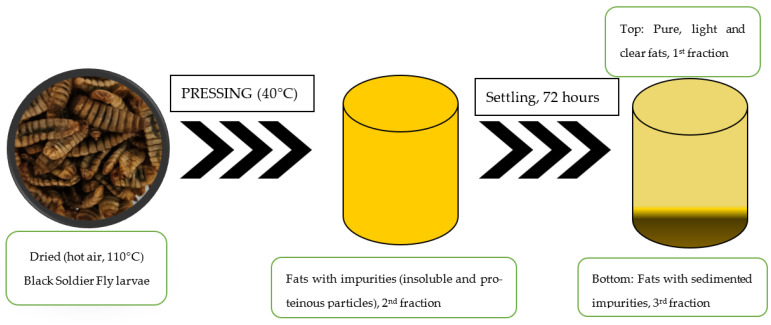
Black Soldier Fly larvae fat production process.

According to Eurostat, every person in the European Union generates 131 kg of food waste, which adds up to nearly 59 million tonnes every year [18]. Food waste is not only an ecological or social problem but also causes a huge financial loss that is estimated to be around 132 billion euros per year. Before now, there has been no opportunity to cut food waste entirely; thus, different solutions, such as biomass reuse, are playing an extremely important part in finding long-term solutions. Black Soldier Fly larvae can be used to convert generated food waste to high nutritional value biomass. The food waste utilisation via Black Soldier Fly larvae would not completely solve the food waste issue, but it can contribute by saving tonnes of valuable biomass and provide nutritious ingredients for human consumption. This research contributes to this knowledge by providing important data on the Black Soldier Fly larvae biomass composition, fat fraction explicit composition and stability data, and by revealing promising findings on the antimicrobial activity of BSF larvae fats. In this research, three different fats were used, which were obtained by pressing the same dried larvae and letting the fats differentiate autonomously. The settling process took 72 h. During this time, the suspended insoluble and proteinous impurities settled to the bottom. After settling, the pure, light and transparent fats (without impurities) were called the 1st (light) fat fraction; the whole fats, right after the pressing process with all insoluble and proteinous impurities, were called the 2nd full fraction (with fats and impurities from the pressing distributed evenly); and lastly, the fats from the lower part of the sedimentation tankage, with all the sedimented impurities and proteinous impurities, were called the 3rd (dark, because of many impurities) fraction. The detailed fractionation process is given in Section 4.1 and Figure 1. The different fat fractions were tested and showed a growth-inhibitory effect against *Candida albicans*, *Bacillus subtilis*, *Staphylococcus aureus* and *Escherichia coli*. The inhibitory effect on *Candida albicans* was confirmed by shelf-life studies using larvae fat-based oleogel in a model food matrix to gain valuable knowledge of the fat’s capability to improve food stability in real-life conditions.

## 2. Results and Discussion

### 2.1. Black Soldier Fly Biomass Composition

The results of the analysis of the pressed and dried BSF larval biomass revealed that the biomass is rich in protein (Table 1), with a protein content of 59.42% in the dry matter and 65.63% in the dry non-fatty matter. For the other components, the total carbohydrate content is 22.11% and the fat content is 8.97%. Carbohydrates without fibre account for 16.81% and are dominated by the exoskeleton substance chitin. Significant amounts of bifidobacteria were found in the BSF larval biomass (Table 1), raising the hypothesis that the larval components may have an effect on the microbiota system.

### 2.2. Black Soldier Fly Larval Biomass Fatty Acid Composition

The total BSF larval biomass fatty acid composition is given in Table 2. The biggest part of the fatty acids (40.93%) consists of lauric fatty acid (C12:0). This fatty acid is a medium-chain acid, which is solid at room temperature and is abundant in coconut and palm kernel oil. Lauric acid is also used for various cosmetic purposes: for example, in the treatment of acne, as it has a growth-inhibiting effect against *Propionibacterium acnes* [19]. In addition, palmitic (C16:0), oleic (C18:1), myristic (C14:0), linolenic (C18:2) and palmitoleic (C16:1) fatty acids are present at significant levels, accounting for 19.11, 17.34, 6.49, 8.79 and 3.89% of the fatty acid content, respectively.

The most important of these fatty acids is linolenic fatty acid, which is classified as an omega-6 fatty acid and is one of the essential fatty acids that humans must obtain from food. It is present in the fat of the BSF biomass at a level of 8.79 g per 100 g of product, which is slightly below the recommended daily dietary intake of 11 g of this fatty acid [20].

Palmitic fatty acid, with a content of 17.34 g per 100 g of BSF larval biomass fat, is the main saturated fatty acid in human fat, accounting for up to 30–35% of the total fatty acids. This fatty acid is very important in the human body, because it has the ability to increase high-density cholesterol without affecting the ratio of the total cholesterol to high-density cholesterol. It is particularly important in the diet of lactating women, as it is involved in the formation of milk [21]. Oleic acid, which is present at a level of 6.49 g per 100 g of BSF larval biomass fatty acids, has anti-inflammatory properties and plays an important role in the activation of different immune response pathways [22]. Myristic fatty acid, with a concentration of 8.79 g per 100 g of BSF larval biomass fat, is used in the food industry as a flavouring ingredient. It is widely found in fats from the plant and animal kingdoms, including in common human foods such as nutmeg. Although the health benefits of this fatty acid have not been established, it is safe for direct consumption [23]. Palmitoleic acid, a monounsaturated fatty acid that is found in abundance in human serum and tissues, particularly in the adipose tissue and liver, is found at a level of 3.89 g per 100 g of BSF larval biomass fat. While trans-palmitoleate is also synthesised in the human body, it is mainly obtained from the diet through ruminant fat and dairy products. Studies are currently underway to investigate whether the stimulation of palmitoleic acid release from adipose tissue, its dietary intake and its supplementation can be used to treat or prevent metabolic disorders [24].

Other fatty acids present in smaller amounts in the BSF larval biomass fat fatty acid profile include stearic fatty acid (C18:0), caproic fatty acid (C10:0), α-linolenic acid (C18:3) and myristoleic acid (C14:1), accounting for 1.73, 0.67, 0.59 and 0.27% of the fatty acid content, respectively. Traces of fatty acids such as pentadecyl (C15:0), margarine (C17:0), oleic (C18:1 trans) and arachidic (C20:0) were detected as well, accounting for 0.06, 0.05, 0.01 and 0.06% of the fatty acid content, respectively.

The most significant of the non-dominant fatty acids is α-linolenic acid. It is classified as an omega-3 fatty acid, which is also essential to include in the human diet. This fatty acid in the fat of the BSF larvae biomass represents approximately 0.59 g per 100 g of product, while a daily intake of 1.4 g of this fatty acid is recommended [20].

Studies show that capric fatty acid exhibits bactericidal and anti-inflammatory properties similar to those of lauric acid. The anti-inflammatory effect may be mediated, in part, by the inhibition of NF-κB activation and the phosphorylation of MAP kinases [24]. Recent research on laboratory mice has led to the hypothesis that myristic fatty acid may have a beneficial effect in treating obesity [25].

The high proportion of saturated fatty acids also means that such fats are solid at room temperature and that the saturated fatty acids are more resistant to oxidation, hydrolysis and other deterioration processes. Although saturated fatty acids are generally considered to be unfavourable to one’s health, the fatty acid profile presented in Table 2 demonstrates that the majority of the fatty acids in the insect larval biomass is composed of health-neutral fatty acids, such as the lauric, palmitic and myristic fatty acids discussed above. Since information about triglycerides in fats is crucial in gaining more knowledge about these novel fat sources and can have an impact on fat digestibility, further researches of Black Soldier Fly larvae fat triglyceride composition is needed.

### 2.3. Black Soldier Fly Larval Biomass Fat Quality, Stability and Safety Parameters

The fat is one of the products of the processing of the BSF larval biomass. Taking into consideration that various fats usually have a shelf-life ranging from 1 to 2 years in food stores, it was decided to determine if BSF larval biomass fat could exhibit a shelf-life of at least one year (>52 weeks) by assessing the stability of the quality indicators. The analysis of its characteristics immediately after production and after storage for 55 weeks (Table 3) demonstrates that it is a valuable and stable product. Furthermore, it contains approximately 9% polyunsaturated fatty acid, which is essential for the human body. The value of the fat is also enhanced by the fact that only trace amounts of trans fatty acids are present in it.

According to the Dietary Reference Values provided by the EFSA [20], alpha-linolenic acid should constitute 0.5% of one’s energy intake, eicosapentaenoic acid and docosahexaenoic acid together should be at least 250 mg/day, and linoleic acid should comprise 4% of the energy intake [20]. Studies on the composition of the fat of the BSF larvae biomass show that almost all of the product is composed of fat (99.82%). However, trans fat only accounts for 0.01% of the fat of the larvae biomass even after 389 days of storage. The significant amount of poly and monosaturated fatty acids indicates that BSF larval fat might be a well-balanced fat source with high stability during storage.

Studies on the composition of the BSF fat have demonstrated that almost all of the product is composed of pure fat (99.82%, Table 3). According to the current requirements of Regulation (EU) 2019/649, the trans fat content must not exceed 2 g/100 g of fat. The data presented in Table 3 reveal that the trans fat content of the fat of the larval biomass is only 0.01%, indicating that the product complies with the above-mentioned requirements both when freshly produced and after 55 weeks of storage. Although the quality of the larval fat is not separately regulated, the requirements for other fats can be used to establish a chemical marker profile for this product.

Commission Regulation (EU) 2019/110 for new oil sources specifies that the unsaponifiable matter shall not exceed 1.0% of the total fatty acids by weight or 10 g/kg, and the saponification value shall not exceed 185–198 mg KOH/g. The requirements of Commission Regulation (EEC) No. 2568/91 for olive oils specify a maximum of 20 meq/kg of fat for the peroxide value, less than 2% for the acidity content and a maximum acidity of up to 3.3 mg KOH/g. Based on the above indicators, it is possible to draw up indicative guidelines for the chemical profile of the BSF larval biomass fat.

BSF fat has a unique saturated fatty acid composition in the sense that the predominant saturated fatty acids are considered to be beneficial to human health and nutrition. Palmitic fatty acid (17.34%) has the ability to increase high-density cholesterol without affecting the ratio of the total cholesterol, while oleic fatty acid (6.49%) is considered to have anti-inflammatory properties, and myristic fatty acid (8.79%) is a valuable flavouring ingredient. It is very important to note that even while being predominant in saturated fatty acids, BSF larval biomass fat has a decent amount (8.79%) of omega-6 (linoleic) fatty acid, of which the daily intake recommendation is 11 g, which is followed by omega-3 (0.59%) with a daily intake recommendation of 1.4 g.

The analytical data and the indicative limits in the regulations show that the peroxide value, oleic acidity, proportion of fatty matter and the acid value do not exceed the quality requirements established for other fats; therefore, it can be concluded that such indicators would be sufficient to ensure the supply of a high-quality product to the market. Only the hydrolysis number exceeds the limits defined in the regulations, which define a maximum limit of 198 mg KOH/g, compared to 217 in the BSF larval biomass fat samples.

### 2.4. Black Soldier Fly Larval Biomass Fat Antimicrobial Activity

According to the literature [26,27], during growth, the larvae can inhibit some micro-organisms and change the composition of the substrate microbiota by secreting antimicrobial substances. The treatment of the larval biomass also involves the use of high temperatures, which is likely to destroy part of the larval microbiota and ensure product safety.

Three BSF larval biomass fat fractions—light, full and dark (as described in Section 4.1)—were analysed. The analysis of the microbiological parameters (Table 4) showed that the main safety parameters were met. The first fraction was considered to be the best quality (pure fat), with the third fraction had the highest prevalence of various bacteria. No *Salmonella* spp., *Listeria monocytogenes* and coliform bacteria were found in any of the three fat fractions even after 55 weeks of storage. Mesophilic lactic acid bacteria in a presumptive bifidobacterial count, sulphite-reducing bacteria (*Clostridia*), mould fungi and yeasts were not detected in the fat, as the thermal process destroyed them and secondary contamination was prevented due to the good hygiene practices followed in the production. Thus, the fat of the BSF biomass does not contain viable food spoiling or pathogenic microorganisms, and in the third fraction, they were only up to 10^5^ due to the higher content of non-fatty impurities.

As the fat fraction of the BSF larval biomass is a newly developed product for the company, the analysis of microbiological contamination provides an insight into whether the fat extraction technology currently in use is sufficient to ensure product safety. In order to ascertain whether microorganisms are able to grow in the fat during long-term storage, the 1st (light) fraction (which made up the biggest part of the total fat) was kept for a period of 55 weeks. As is mentioned in Section 4.1, the 2nd and 3rd fractions had impurities. The 2nd fraction was made up totally from fat right after pressing and included different impurities (insoluble parts and proteinous particles), while the 3rd fraction had a high amount of impurities concentrated in this fraction after the pressing and settling process. Only the 1st fraction contained pure Black Soldier Fly larvae fat. During the shelf-life and stability studies with the 2nd and 3rd fractions, the stability or sensitivity to oxidation or microbial growth could be caused not only by the fat but also due to the nature of the impurities. Since the aim of the research was to gain knowledge about the fats, only pure fat—the 1st fraction—was selected for the long period of shelf-life stability studies.

The analysis revealed that the BSF larval biomass fat light fraction did not induce the growth of spoilers and pathogens. This finding is very important for the development of food matrices that incorporate certain BSF fat fractions.

The antimicrobial properties of proteins and fats were evaluated by the agar diffusion method. The protein fraction was included as a reference sample to prove that the fat fraction has a higher capability of inhibiting microorganism growth. For the evaluation of the antimicrobial efficiency of the protein and fat fractions of BSF larvae, 10% ethanolic solutions of the four different fractions were made (Table 5). Gram-positive cultures of *Bacillus subtilis* and *Staphylococcus aureus* and Gram-negative cultures of *Escherichia coli, Pseudomonas aeruginosa,* and *Salmonella typhimurium* and a culture of the pathogenic yeast *Candida albicans*, belonging to the microscopic fungi, were used for the tests. The data show that the bacteria are more strongly affected by the 3rd (dark) fraction of BSF larval biomass fat, with larger zones of inhibition, but the Gram-positive bacteria *Bacillus subtilis* and *Staphylococcus aureus* are more sensitive, with inhibition zones with diameters of 16.5 ± 0.5 and 12.5 ± 1.5 mm, respectively. The 2nd full fat fraction of BSF fat is the least effective, while Gram-negative cultures of *Escherichia coli, Pseudomonas aeruginosa,* and *Salmonella typhimurium* proved to be totally resistant.

All three fat fractions inhibited the growth of the yeast *Candida albicans* with inhibition zones of 10.5 ± 0.7, 12.5 ± 0.9 and 12.5 ± 0.9 mm in diameter, respectively. The protein fraction solution has no antimicrobial properties and inhibits only one bacterium, *Bacillus subtilis*, with an inhibition zone diameter of 12.5 ± 1.5 mm. The data obtained suggest that BSF larval fat demonstrates antimicrobial properties that could improve the microbiological safety and stability of the microbiological parameters in food matrices.

### 2.5. Stability Studies of BSF Larval Fat in a Model Food Matrix

As detailed in Section 4.4, plant-based model matrixes were created including different amounts of BSF larval fat oleogels, which were created by mixing the larval fat with vegetable (rapeseed) oil. Since in BSF, larval fats showed promising oxidative stability, antibacterial and antifungal activity, research on the model food matrix was conducted.

Model food matrixes with 30 and 50% BSF larvae fat, containing oleogel (OG30 and OG 50, respectively) in the sample (Table 6 and Table 7, respectively), were produced, and the composition and dynamics of the quality and safety parameters were monitored during the test period.

Model food matrixes were also made with the addition of the BSF oleogels (BSF larval fat (1st) blended with rapeseed oil 50:50 (OG50) and 30:70 (OG30)) to investigate if a smaller amount of the BSF larval fat would still improve the stability and safety of the food matrix. The plant-based model matrix analytical data revealed that in this case, only a third of the total fat in the matrix was saturated fatty acids. It was also observed that the addition of the BSF larval fat had the property of liquefying the matrix (the prepared sample was not so firm as the control), which may be considered to be a disadvantage in the use of the product, as the use of an additional binder may be required. When the oleogels were produced by mixing the BSF fat (1st fraction) with vegetable oil, this property became less pronounced. For a further investigation of this phenomena, a texture analysis of the prepared matrixes is needed for the BSF larval biomass fat’s technological properties.

The produced plant-based model matrixes were stored for a period of 45 days. The chemical and microbiological analyses for the matrixes were repeated after 30 and 45 days. The increase in the peroxide value, acidity and the number of acids during the test period were determined. It was concluded that during storage, the fat underwent a degradation processes and that the vacuum packaging did not protect the fat from environmental influences.

The microbiological indicators also changed with the total number of microorganisms increasing from an initial 1.6 × 10^2^ to 1.6 × 10^6^ after 30 days and to 1.6 × 10^2^ after 45 days. The model food matrix was unfavourable for coliform bacteria, whose numbers decreased from 7 CFU/g to less than 1 during the storage period. Mesophilic lactic acid bacteria and presumptive bifidobacteria were not detected in the matrixes with OG30 and OG50 at the beginning of the study, but after 45 days of storage at a low temperature (+4 °C), they were already present with a higher level in the matrix with OG30. The number of moulds and fungi in the food matrix decreased to undetectable levels in the matrix with OG50 during the 30-day exposure, while the number of microscopic fungi in the matrix with OG30 decreased during the whole storage period.

## 3. Conclusions

The biomass composition of the BSF *Hermetia Illucens* larvae contained certain amounts of biologically active substances, and the physicochemical and microbiological characteristics of the fat were investigated. The results of the study revealed that the Black Soldier Fly larvae fat is predominantly composed of lauric fatty (40.93%), followed by palmitic, oleic, myristic, linolenic and palmitoleic fatty acids, accounting for 19.11, 17.34, 6.49, 8.79 and 3.89% of the fatty acid content, respectively. The oxidative stability of the BSF larvae biomass was investigated by assessing the temporal dynamics of the fat oxidation products—peroxides. A 55-week shelf-life study showed that the pure fat fraction (named the 1st fraction) presented stability according to the acidity (as oleic acid), as in 55 weeks, it increased 0.1% in the fat, the acid value increased 0.19 mg KOH/g of fat, the unsaponifiable matter increased 1.4 g/kilogram fat and the iodine value decreased 1.24 g/100 g fat. Since peroxides have a tendency to degrade during their shelf-life and produce secondary oxidation products, TBARS (thiobarbituric acid reactive substances), an analysis should be included in future research.

The different fat fractions were also tested for antimicrobial activity. They showed an efficiency against *Candida albicans* (the inhibition zone varied from 10.5 (1st fraction) to 12.5 (2nd and 3rd fraction) mm), *Bacillus subtilis* (from 12.5 (1st fraction) to 16.5 (3rd fraction) mm), *Staphylococcus aureus* (12.5 mm, 3rd fraction) and *Escherichia coli* (10.0 mm, 3rd fraction). Since the 1st and 3rd fractions, which had different amounts of proteinous impurities, both showed an antimicrobial activity, a protein fraction was included in the antimicrobial activity determination, but it showed activity only against *B. subtilis* (10.0 mm diameter). The inhibitory effect on *Candida albicans* was confirmed by shelf-life studies using larvae fat-based oleogel in a model food matrix.

Even though the BSF larval biomass fat is dominated by saturated fatty acids, a qualitative fatty acid analysis showed that the saturated fatty acids in the product have a beneficial effect and are nutritionally valuable in the human diet. This indicates that a food product enriched with larval biomass will not only have a high nutritional and biological value, but it also might have a long shelf life. It has been experimentally established that the fat of BSF larvae has antimicrobial properties and inhibits the growth of Gram-positive bacteria and yeasts. The results of the study lead to the conclusion that a food matrix enriched with the fatty biomass of the larvae would have a preventive effect against infections caused by yeast fungi (e.g., gastric and intestinal candidiasis).

## 4. Materials and Methods

Black Soldier Fly larvae can be used as one of the tools to create a circular economy model in areas such as waste management and the treatment of industrial by-products to produce high added-value food grade ingredients. This article is aimed at providing ready-to-use knowledge that can be easily adapted for use in the waste management industry. The obtained data give a broad view of the potential benefits and risks of using Black Soldier Fly larvae fats for human consumption. A shelf-life study was conducted with a pure larvae fat fraction (in the text named the 1st fraction) to show the stability of pure Black Soldier Fly larvae fats. For the purpose of testing the fats in real-life food matrixes, a high-humidity, plant-based sample was used to represent most short shelf-life products.

To provide reproducible results not only in a scientific environment but also ensure reproducibility and adaptability directly in the food industry, most of the methodologies used in this study were based on international standards that can be repeated and followed in any adequate laboratory.

### 4.1. Preparation of the Different BSF Larval Fat Fractions

The fat was separated from the total BSF larval biomass by pressing, and the production flow is shown in Figure 1. When the BSF larvae reached an optimal size, they were frozen, blanched and dried using hot (110 °C) air. The dried larvae were then pressed at 40 °C until the fat was removed from the mass. The fat was left to settle autonomously in order to obtain pure fat without impurities. The settling process was carried out at a temperature of 40 °C for 72 h. During the settling process, the suspended protein impurities and insoluble biomass residues settled to the bottom of the container. After the settling process, a dark fat fraction was formed at the bottom of the container, which contained fat with a large amount of settled impurities (proteins and insoluble biomass residues). This fraction was removed by sedimentation until the upper, light and transparent fat fraction was reached, which was without impurities. The middle fraction was the fat obtained after pressing. It was not subject to sedimentation and consisted of both light pure fat and impurities.

1st (light) fraction—pure fats after the pressing and sedimentation process.
2nd (full) fraction—fats after pressing, including proteins and other biomass residues.
3rd (dark) fraction—fats after pressing and sedimentation from the lower part with sedimented proteins and insoluble biomass residues.

### 4.2. Methods of the Microbiological Analysis

#### 4.2.1. Total Number of Microorganisms

The total number of microorganisms was determined by the Petri dish method using Plate Count Agar (LAB M), according to LST EN ISO 4833–1:2013 “Microbiology of food and feed. General method. Method for counting colonies at 30 °C”.

#### 4.2.2. Coliform Count Determination

The determination of individual bacteria was performed as a coliform count at 37 °C, CFU/g, detection of Salmonella at 25 g, total number of mesophilic lactic acid bacteria, CFU/g, number of presumptive bifidobacteria, CFU/g, total number of sulphite-reducing bacteria (clostridia), CFU/g, number of yeasts, CFU/g, number of moulds, CFU/g, and detection of monocytogenic listeria (Listeria monocytogenes) at 37 °C at 25 g, using the following methods of analysis: LST EN ISO 11133:2014 “Microbiology of food, feed and water”.

#### 4.2.3. *Escherichia coli* Determination

LST ISO 16649–2:2002 “Microbiology of food and feed—Colony counting in food and feed General method for the enumeration of β–glucuronidase–producing enteric rods (*Escherichia coli*)—Methods and methods Part 2: Methods for the determination of Escherichia coli Method for counting colonies at 44 °C using 5–bromo–4–chloro–3–indolyl β–D–glucuronide”.

#### 4.2.4. *Listeria monocytogenes* Determination

LST EN ISO 11290–1:2017 “Microbiology of the food chain—General method for the detection and enumeration of Listeria monocytogenes and other Listeria species—Tests for the detection and enumeration of Listeria monocytogenes and other Listeria species Part 1. Detection method”.

#### 4.2.5. Presumptive Bifidobacteria Determination

LST ISO 29981:2010 “Dairy products—Part 1: Methods and methods Enumeration of presumptive bifidobacteria—Dairy products—Methods and methods Method for counting colonies at 37 °C (identical to ISO 29981:2010)”.

#### 4.2.6. Black Soldier Fly Larvae Antibacterical Activity Determination

Preparation of the BSF larvae biomass fractions for antibacterial activity: protein and fat fractions of BSF larvae 10% ethanolic solutions of the 4 different fractions were made (protein fraction, 1st (light) fat fraction, 2nd (full) fat fraction and 3rd (dark) fat fraction). Pure solvent (10% ethanol solution) was used as a control.

The agarised media were inoculated with different types of microorganisms (*Bacillus subtilis, Staphylococcus aureus Escherichia coli, Pseudomonas aeruginosa*, *Salmonella typhimurium,* and *Candida albicans*). On the hardened medium, wells (8 mm in diameter) were cut out and filled with the testing samples of the fat and protein solution (50 µL each well, 6 wells per plate). The plates were incubated with the thermostat at 30 ± 2 °C for 5 days. Every 24 h, the growth of the isolate was measured with a ruler in two perpendicular directions until the growth of the test isolate on the control plate reached the edge of the plate.

### 4.3. Methods of the Chemical Analysis

#### 4.3.1. Moisture Content Determination

Moisture and volatile matter content, % (LST EN ISO 662:2016, p.8). Moisture content, % (LST ISO 1442:2000).

#### 4.3.2. Fat Content Determination

Total fat content,% saturated fatty acids, % monounsaturated fatty acids, % polyunsaturated fatty acids, % trans fatty acids, % (LST ISO 1443:2000; LST EN ISO 12966–1:2015; LST EN ISO 12966–2:2017). Fatty acid composition, % of total fatty acids: (LST EN ISO 12966–1:2015 LST EN ISO 12966–2:2017).

#### 4.3.3. Fat Quality Parameters Determination

Acidity by oleic acid (free fatty acid content), %; Acid value, mg KOH/g (LST EN ISO 660:2020, p.9.1). Unsaponifiable matter content, g/kg (LST EN ISO 3596:2003). Insoluble impurities, % (LST EN ISO 663:2009). Iodine content, g I/100 g (LST EN ISO 3961:2013). Phosphorus content, mg/kg (AOAC 970.39, 15th edition). Hydrolysis number, mg KOH/g (ISO 3657:2020). Peroxide value, meq/kg of fat (LST EN ISO 3960:2017). Fat acidity (free fatty acid content) based on oleic acid, % of fat acid number, mg KOH/g fat (LST EN ISO 660:2020, p.9.1).

#### 4.3.4. Histamine Content Determination

Histamine content, mg/kg (LST EN ISO 19343:2017; “Determination of histamine content. Method for effective liquid chromatography”).

#### 4.3.5. Nutritional Value Determination

Determination of pH 10% (LST ISO 1842:1997). Nitrogen content, % (LST ISO 937:2000). Protein content, % (calculated by multiplying the nitrogen content by a factor of 6.25 (Regulation (EU) No. 1169/2011 of the European Parliament and of the Council, Annex I, p. 10). Total ash content, % (LST ISO 936:2000). Fibrous matter content, % (AOAC 985.29, 1990). Total carbohydrate content, %, (carbohydrate content (excluding fibre), %, calculated from the difference according to “Composition of Foodstuffs”, 2002, Vilnius); Energy value per 100 g, kJ (calculation according to Regulation (EU) No. 1169/2011, Annex XIV).

### 4.4. Preparation of the Model Food Matrix

To investigate the Black Soldier Fly larvae fat’s capacity to improve microbiological stability in a composite food matrix, a representative food sample was selected. Since products with high humidity levels tend to be more susceptible to microorganism growth, a neutral plant-based formulation was selected. This formulation gave the impression of a high moisture product, which at the beginning had low levels of microbiological indicators and had the capacity to support growth (a high amount protein with a decent amount of carbohydrates and water). The plant-based model food matrix was prepared according to the recipe given in Table 8. The recipe was created to mimic a health-beneficial, rich in protein novel food matrix. Each sample weighted 150 g and was formed in a round, flat shape (3 cm high) and was vacuum-sealed in a bag mostly used for this type of food. Each sample line had 6 parallel samples for the needed repetitions.

### 4.5. Statistical Analysis

GraphPad Prism (ver. 8.0.1) was used for the statistical data processing. A one-way ANOVA test was performed to indicate significant differences where needed.

## Figures and Tables

**Table 1:** Characterisation of the pressed and dried BSF larval biomass.

Indicator	Results
pH, 10% solution	6.46
Moisture, %	5.25
Fat content, %	8.97
Nitrogen content, %	9.00
Protein content, %	56.30
Protein content in dry matter, %	59.42
Protein content in dry non–fat matter, %	65.63
Total ash content, %	7.37
Fibre content, %	5.30
Total carbohydrates, %	22.11
Presumptive bifidobacteria count, CFU/g	8.26 × 10^7^

**Table 2:** BSF larval biomass fat fatty acid composition.

Fatty Acid	Result, % from Total Fat Amount	Fatty Acid	Result, % from Total Fat Amount
C_4:0_	0.00	C_18:3W6_	0.00
C_6:0_	0.00	C_18:3trans_	0.00
C_8:0_	0.00	C_18:4W3_	0.00
C_10:0_	0.67	C_20:0_	0.06
C_12:0_	40.93	C_20:1_	0.00
C_13:0_	0.00	C_20:2_	0.00
C_14:0_	6.49	C_20:3n3W3_	0.00
C_14:1_	0.27	C_20:3n6W6_	0.00
C_15:0_	0.06	C_20:4W6_	0.00
C_15:1_	0.00	C_20:5_ EPA	0.00
C_16:0_	19.11	C_21:0_	0.00
C_16:1_	3.89	C_22:0_	0.00
C_16:2W6_	0.00	C_22:1_	0.00
C_17:0_	0.05	C_22:2W6_	0.00
C_17:1_	0.00	C_22:4W6_	0.00
C_18:0_	1.73	C_22:5_ DPA	0.00
C_18:1_	17.34	C_22:6_ DHA	0.00
C_18:1trans_	0.01	C_23:0_	0.00
C_18:2W6_	8.79	C_24:0_	0.00
C_18:2trans_	0.00	C_24:1_	0.00
C_18:3W3_	0.59	C_24:6_	0.00

**Table 3:** BSF larval biomass fat quality and stability indicators.

Indicator	Results	After 55 Weeks
Moisture and volatile matter content, %	0.18	0.14
Fat content, %, of which:	99.82	99.84
Saturated fatty acids, %	68.98	69.99
Monounsaturated fatty acids, %	21.46	20.87
Polyunsaturated fatty acids, %	9.36	8.98
Trans fatty acids, %	0.01	0.01
Peroxide value, meq/kg of fat	1.1	3.0
Acidity (free fatty acid content) as oleic acid, % in fat	0.75	0.85
Acid value, mg KOH/g of fat	1.50	1.69
Unsaponifiable matter, g/kg	9.8	11.2
Insoluble impurities content, %	0.06	0.08
Iodine value, g/100 g	35.5	34.26
Phosphorus content, mg/kg	1357	2398
Saponification value, mg KOH/g	217	223

**Table 4:** BSF larval biomass fat fraction microbiological indicators.

Indicator	1st (Light) Fraction	2nd (Full) Fraction	3rd (Dark) Fraction	1st (Light) Fraction after 55 Weeks
Total microorganism count, CFU/g	yes. but <4.0 × 10^1^	4.0 × 10^1^	1.4 × 10^5^	5.0 × 10^1^
Coliform bacteria count, CFU/g	<1.0 × 10^1^	<1.0 × 10^1^	<1.0 × 10^1^	<1.0 × 10^1^
Detection of *Salmonella* spp., in 25 g	Not detected	Not detected	Not detected	Not detected
Detection of *Listeria monocytogenes* at 37 °C, in 25 g	Not detected	Not detected	Not detected	Not detected
Total mesophilic lactic acid bacteria count, CFU/g	<1.0 × 10^1^	<1.0 × 10^1^	<1.0 × 10^1^	<1.0 × 10^1^
Presumptive bifidobacteria count, CFU/g	<1.0 × 10^1^	<1.0 × 10^1^	<1.0 × 10^1^	<1.0 × 10^1^
Total sulphite-reducing bacteria (*Clostridia*) count, CFU/g	<1.0 × 10^1^	<1.0 × 10^1^	<1.0 × 10^1^	<1.0 × 10^1^
Mould count, CFU/g	<1.0 × 10^1^	<1.0 × 10^1^	<1.0 × 10^1^	Yes. but <4.0 × 10^1^
Yeast count, CFU/g	<1.0 × 10^1^	<1.0 × 10^1^	<1.0 × 10^1^	<1.0 × 10^1^

**Table 5:** Evaluation of the antimicrobial efficiency of different fractions of insect larval biomass proteins and fats.

Sample	Suppression Zone Diameter, mm with Test Cultures					
	*Bacillus subtilis*	*Staphylococcus aureus*	*Escherichia coli*	*Pseudomonas aeruginosa*	*Salmonella typhimirium*	*Candida albicans*
1st (light) fat fraction	12.5 ± 1.5	0	0	0	0	10.5 ± 0.7
2nd (full) fat fraction	0	0	0	0	0	12.5 ± 0.9
3rd (dark) fat fraction	16.5 ± 0.5	12.5 ± 1.5	10.0 ± 0.0	0	0	12.5 ± 0.9
Protein fraction	10.0 ± 0.3	0	0	0	0	0

**Table 6:** Evaluation of the stability properties of the plant-based model matrix with OG50 (BSF larval fat: rapeseed oil) during its shelf-life at +4 °C.

Indicator	Preparation Day	After 30 Days	After 45 Days
Fat content, %, of which:	18.2	18.18	18.2
Saturated fatty acids, %	6.89	6.86	6.90
Monounsaturated fatty acids, %	7.86	7.80	7.71
Polyunsaturated fatty acids, %	3.44	3.52	3.60
Trans fatty acids, %	0.01	0.00	0.00
Histamine content, mg/kg	<5	<5	<5
Peroxide value, meq/kg of fat	7.5	7.8	8.1
Acidity (free fatty acid content) as oleic acid, % in fat	2.1	2.8	3.0
Acid value, mg KOH/g of fat	4.1	5.2	5.9
Total microorganism count, CFU/g	1.6 × 10^2^	1.6 × 10^6^	1.1 × 10^7^
Coliform bacteria count, CFU/g	<1.0 × 10^1^	5.0 × 10^1^	<1.0 × 10^1^
Detection of *Salmonella* spp., in 25 g	Not detected	Not detected	Not detected
Detection of *Listeria monocytogenes* at 37 °C, in 25 g	Not detected	Not detected	Not detected
Total mesophilic lactic acid bacteria count, CFU/g	8.0 × 10^1^	<1.0 × 10^1^	8.0 × 10^1^
Presumptive bifidobacteria count, CFU/g	<1.0 × 10^1^	<1.0 × 10^1^	3.5 × 10^3^
Total sulphite-reducing bacteria (*Clostridia*) count, CFU/g	<1.0 × 10^1^	<1.0 × 10^1^	<1.0 × 10^1^
Yeast count, CFU/g	<1.0 × 10^1^	3.4 × 10^4^	<1.0 × 10^1^
Mould count, CFU/g	4.7 × 10^2^	Yes, but <4.0 × 10^1^	Yes, but <4.0 × 10^1^

**Table 7:** Evaluation of the stability properties of the plant-based model food matrix with OG30 (30% BSF larval biomass fat: 70% rapeseed oil) during its shelf-life at +4 °C.

Indicator	Preparation Day	After 30 Days	After 45 Days
Fat content, % of which:	20.3	18.9	17.9
Saturated fatty acids, %	4.75	4.53	4.21
Monounsaturated fatty acids, %	10.78	9.68	9.30
Polyunsaturated fatty acids, %	4.76	4.62	4.38
Trans fatty acids, %	0.01	0.00	0.01
Histamine content, mg/kg	<5	<5	<5
Peroxide value, meq/kg of fat	15.5	14.9	14.7
Acidity (free fatty acid content) as oleic acid, % in fat	2.6	2.4	2.1
Acid value, mg KOH/g of fat	5.2	4.5	4.2
Total microorganism count, CFU/g	8.8 × 10^2^	1.9 × 10^7^	1.9 × 10^7^
Coliform bacteria count, CFU/g	7.0 × 10^1^	yes, but <4.0 × 10^1^	<1.0 × 10^1^
Detection of *Salmonella* spp., in 25 g	Not detected	Not detected	Not detected
Detection of *Listeria monocytogenes* at 37 °C, in 25 g	Not detected	Not detected	Not detected
Total mesophilic lactic acid bacteria count, CFU/g	1.1 × 10^2^	<1.0 × 10^1^	1.0 × 10^3^
Presumptive bifidobacteria count, CFU/g	<1.0 × 10^1^	<1.0 × 10^1^	7.8 × 10^3^
Total sulphite-reducing bacteria (*Clostridia*) count, CFU/g	<1.0 × 10^1^	<1.0 × 10^1^	<1.0 × 10^1^
Yeast count, CFU/g	9.0 × 10^1^	2.2 × 10^4^	<1.0 × 10^1^
Mould count, CFU/g	1.1 × 10^2^	1.7 × 10^2^	7.0 × 10^1^

**Table 8:** Recipes of the plant-based model matrix with OG30 and OG50 (BSF larval biomass fat: rapeseed oil).

Ingredient	Matrix 30:70 (OG30)	Matrix 50:50 (OG50)
Pea protein texturate	27.1%	27.1%
Water	54.2%	54.2%
Pea fibre	1.9%	1.9%
Rapeseed oil	11.76%	8.4%
BSF larval biomass fat	5.04%	8.4%

## Data Availability

Not applicable.

## References

[1] Bessa L.W., Pieterse E., Marais J., Hoffman L.C. (2020). Why for feed and not for human consumption? The black soldier fly larvae. Compr. Rev. Food Sci. Food Saf..

[2] Koch J.A., Bolderdijk J.W., Van Ittersum K. (2021). Disgusting? No, just deviating from internalized norms. Understanding consumer skepticism toward sustainable food alternatives. J. Environ. Psychol..

[3] Singh A., Kumari K. (2019). An inclusive approach for organic waste treatment and valorisation using Black Soldier Fly larvae: A review. J. Environ. Manag..

[4] Elhag O., Zhang Y., Xiao X., Cai M., Zheng L., Jordan H.R., Tomberlin J.K., Huang F., Yu Z., Zhang J. (2022). Inhibition of Zoonotic Pathogens Naturally Found in Pig Manure by Black Soldier Fly Larvae and Their Intestine Bacteria. Insects.

[5] Li X., Dong Y., Sun Q., Tan X., You C., Huang Y., Zhou M. (2022). Growth and Fatty Acid Composition of Black Soldier Fly *Hermetia illucens* (Diptera: Stratiomyidae) Larvae Are Influenced by Dietary Fat Sources and Levels. Animals.

[6] Marusich E., Mohamed H., Afanasev Y., Leonov S. (2020). Fatty Acids from *Hermetia illucens* Larvae Fat Inhibit the Proliferation and Growth of Actual Phytopathogens. Microorganisms.

[7] Smets R., Goos P., Claes J., Van Der Borght M. (2021). Optimisation of the lipid extraction of fresh black soldier fly larvae (*Hermetia illucens*) with 2-methyltetrahydrofuran by response surface methodology. Sep. Purif. Technol..

[8] Papargyropoulou E., Lozano R., Steinberger J.K., Wright N., Ujang Z.B. (2014). The food waste hierarchy as a framework for the management of food surplus and food waste. J. Clean. Prod..

[9] El-Dakar M.A., Ramzy R.R., Ji H., Plath M. (2020). Bioaccumulation of residual omega-3 fatty acids from industrial Schizochytrium microalgal waste using black soldier fly (*Hermetia illucens*) larvae. J. Clean. Prod..

[10] Janssen R.H., Vincken J.-P., Van Den Broek L.A.M., Fogliano V., Lakemond C.M.M. (2017). Nitrogen-to-Protein Conversion Factors for Three Edible Insects: *Tenebrio molitor*, *Alphitobius diaperinus*, and *Hermetia illucens*. J. Agric. Food Chem..

[11] Van Huis A. (2013). Potential of Insects as Food and Feed in Assuring Food Security. Annu. Rev. Entomol..

[12] Jones L.D., Cooper R.W., Harding R.S. (1972). Composition of Mealworm Tenebrio molitor Larvae. J. Zoo Anim. Med..

[13] Yi L., Lakemond C.M.M., Sagis L.M.C., Eisner-Schadler V., Van Huis A., Van Boekel M.A.J.S. (2013). Extraction and characterisation of protein fractions from five insect species. Food Chem..

[14] Zhao X., Vázquez-Gutiérrez J.L., Johansson D.P., Landberg R., Langton M. (2016). Yellow Mealworm Protein for Food Purposes—Extraction and Functional Properties. PLoS ONE.

[15] Heuel M., Sandrock C., Leiber F., Mathys A., Gold M., Zurbrügg C., Gangnat I.D.M., Kreuzer M., Terranova M. (2021). Black soldier fly larvae meal and fat can completely replace soybean cake and oil in diets for laying hens. Poult. Sci..

[16] Harlystiarini H., Mutia R., Wibawan I.W.T., Astuti D.A. (2019). In Vitro Antibacterial Activity of Black Soldier Fly (*Hermetia illucens*) Larva Extracts Against Gram-Negative Bacteria. Bul. Peternak.

[17] Hadj Saadoun J., Luparelli A.V., Caligiani A., Macavei L.I., Maistrello L., Neviani E., Galaverna G., Sforza S., Lazzi C. (2020). Antimicrobial Biomasses from Lactic Acid Fermentation of Black Soldier Fly Prepupae and Related By-Products. Microorganisms.

[18] Eurostat (2023). Food Waste and Food Waste Prevention—Estimates. https://ec.europa.eu/eurostat/statistics-explained/index.php?title=Food_waste_and_food_waste_prevention_-_estimates.

[19] Nakatsuji T., Kao M.C., Fang J.-Y., Zouboulis C.C., Zhang L., Gallo R.L., Huang C.-M. (2009). Antimicrobial Property of Lauric Acid Against Propionibacterium Acnes: Its Therapeutic Potential for Inflammatory Acne Vulgaris. J. Investig. Dermatol..

[20] EFSA Dietary Reference Values for the EU Released by EFSA. https://multimedia.efsa.europa.eu/drvs/index.htm.

[21] Agostoni C., Moreno L., Shamir R. (2016). Palmitic Acid and Health: Introduction. Crit. Rev. Food Sci. Nutr..

[22] Sales-Campos H., de Souza P.R., Peghini B.C., da Silva J.S., Cardoso C.R. (2013). An overview of the modulatory effects of oleic acid in health and disease. Mini Rev. Med. Chem..

[23] Burdock G.A., Carabin I.G. (2007). Safety assessment of myristic acid as a food ingredient. Food Chem. Toxicol..

[24] Frigolet M.E., Gutiérrez-Aguilar R. (2017). The Role of the Novel Lipokine Palmitoleic Acid in Health and Disease. Adv. Nutr..

[25] Huang W.-C., Tsai T.-H., Chuang L.-T., Li Y.-Y., Zouboulis C.C., Tsai P.-J. (2014). Anti-bacterial and anti-inflammatory properties of capric acid against Propionibacterium acnes: A comparative study with lauric acid. J. Dermatol. Sci..

[26] Quan L.-H., Zhang C., Dong M., Jiang J., Xu H., Yan C., Liu X., Zhou H., Zhang H., Chen L. (2020). Myristoleic acid produced by enterococci reduces obesity through brown adipose tissue activation. Gut.

[27] Gold M., Von Allmen F., Zurbrügg C., Zhang J., Mathys A. (2020). Identification of Bacteria in Two Food Waste Black Soldier Fly Larvae Rearing Residues. Front. Microbiol..

